# Zmiz1 is a novel regulator of brain development associated with autism and intellectual disability

**DOI:** 10.3389/fpsyt.2024.1375492

**Published:** 2024-04-15

**Authors:** Rajan K. C., Alina S. Tiemroth, Abbigail N. Thurmon, Stryder M. Meadows, Maria J. Galazo

**Affiliations:** ^1^Department of Cell and Molecular Biology, Tulane University, New Orleans, LA, United States; ^2^Tulane Brain Institute, Tulane University, New Orleans, LA, United States

**Keywords:** Zmiz1, *de novo* mutations, neurodevelopment, neurodevelopmental disorders, autism

## Abstract

Neurodevelopmental disorders (NDDs) are a class of pathologies arising from perturbations in brain circuit formation and maturation with complex etiological triggers often classified as environmental and genetic. Neuropsychiatric conditions such as autism spectrum disorders (ASD), intellectual disability (ID), and attention deficit hyperactivity disorders (ADHD) are common NDDs characterized by their hereditary underpinnings and inherent heterogeneity. Genetic risk factors for NDDs are increasingly being identified in non-coding regions and proteins bound to them, including transcriptional regulators and chromatin remodelers. Importantly, *de novo* mutations are emerging as important contributors to NDDs and neuropsychiatric disorders. Recently, *de novo* mutations in transcriptional co-factor Zmiz1 or its regulatory regions have been identified in unrelated patients with syndromic ID and ASD. However, the role of Zmiz1 in brain development is unknown. Here, using publicly available databases and a Zmiz1 mutant mouse model, we reveal that Zmiz1 is highly expressed during embryonic brain development in mice and humans, and though broadly expressed across the brain, Zmiz1 is enriched in areas prominently impacted in ID and ASD such as cortex, hippocampus, and cerebellum. We investigated the relationship between Zmiz1 structure and pathogenicity of protein variants, the epigenetic marks associated with Zmiz1 regulation, and protein interactions and signaling pathways regulated by Zmiz1. Our analysis reveals that Zmiz1 regulates multiple developmental processes, including neurogenesis, neuron connectivity, and synaptic signaling. This work paves the way for future studies on the functions of Zmiz1 and highlights the importance of combining analysis of mouse models and human data.

## Introduction

Brain development requires a precisely orchestrated sequence of events, including neurogenesis, neuronal migration, differentiation, cell death, synaptogenesis, and myelination. These processes collectively shape brain architecture, connectivity, and function. Disruptions in neurodevelopmental processes result in a wide variety of neurodevelopmental disorders (NDDs), such as autism spectrum disorders (ASD), attention deficit hyperactivity disorders (ADHD), and intellectual disability (ID) (1, 2). NDDs profoundly affect the developmental trajectory of key brain structures such as the cortex, striatum, hippocampus, thalamus, hypothalamus, and cerebellum resulting in a wide range of cognitive and behavioral deficits impacting social interactions, communication, motor skills, learning, and memory (3, 4).

NDDs are caused by a complex interplay of genetic, epigenetic, and environmental risk factors, with genes and regulatory elements such as transcription factors (TFs), coregulators, and chromatin remodelers playing pivotal roles in its pathogenesis (3). Importantly, *de novo* mutations in transcriptional regulators are emerging as important contributors to NDDs. Between 40-50% of individuals with NDDs are thought to harbor a causative *de novo* variant (5–7). Specifically in ASD, 50% of cases are attributed to common genetic variants, including around 40% *de novo* mutations (5, 8). Understanding the emerging role of *de novo* mutations in transcriptional regulators is critical to advance our knowledge of NDD pathogenesis and improve therapeutic interventions for neurological disorders.

Recently*, de novo* mutations in the coding and regulatory regions of transcriptional coregulator and chromatin remodeler, Zinc Finger MIZ-Type Containing 1 (*ZMIZ1*) have recently been associated with various NDDs, including ID, ASD, and ADHD (9, 10). However, the role of Zmiz1 in brain development and associated pathologies has not been investigated. A comprehensive study of Zmiz1 expression in the developing brain, the pathogenic risk represented by *ZMIZ1 de novo* variants, and the signaling pathways potentially regulated by Zmiz1 is necessary to illuminate how these pathogenic mutations impact early brain development and unveil the etiological nature of Zmiz1-associated neurodevelopmental disorders.

Here we use publicly available resources and a conditional cortex-specific *Zmiz1*-knockout mouse model to investigate the role of Zmiz1 in brain development. Our studies reveal that *Zmiz1* is highly expressed during embryonic brain development in both mice and humans. Although *Zmiz1* is broadly expressed in different cell types throughout the brain, it is notably enriched in brain regions highly impacted in ASD and ID, such as the neocortex, hippocampus, and cerebellum. Analysis of ZMIZ1 structure and mutation load suggests important functions in transcriptional control, formation of multi-molecular complexes, and high intolerance to potentially pathogenic variants. Molecularly, ZMIZ1 is strongly associated with open chromatin marks such as H3K9ac, H3K27ac, H3K4me3, and H3K4me2, which indicate functions as a transcriptional co-activator and chromatin remodeler. Transcriptomic profiling suggests that the loss of *Zmiz1* function impairs cortical neurogenesis, neuron differentiation, and synaptic signaling gene expression profiles. Interestingly, *Zmiz1* displays differential expression in neuropil compared to soma, with cytoplasmic expression in neurons of unknown function. Importantly, the ZMIZ1 interaction network includes genes associated with ASD and other NDDS. This study lays the foundation for future work on the function of Zmiz1 in neurodevelopment, highlights the impact of *de novo* mutations in neurodevelopmental disorders, and underscores the importance of combining analyses of mouse models and human data to advance our understanding of neurodevelopment.

## Results

### Zmiz1 is broadly expressed in human and mouse embryonic brain, but enriched in specific regions and cell types

**Figure 1:**
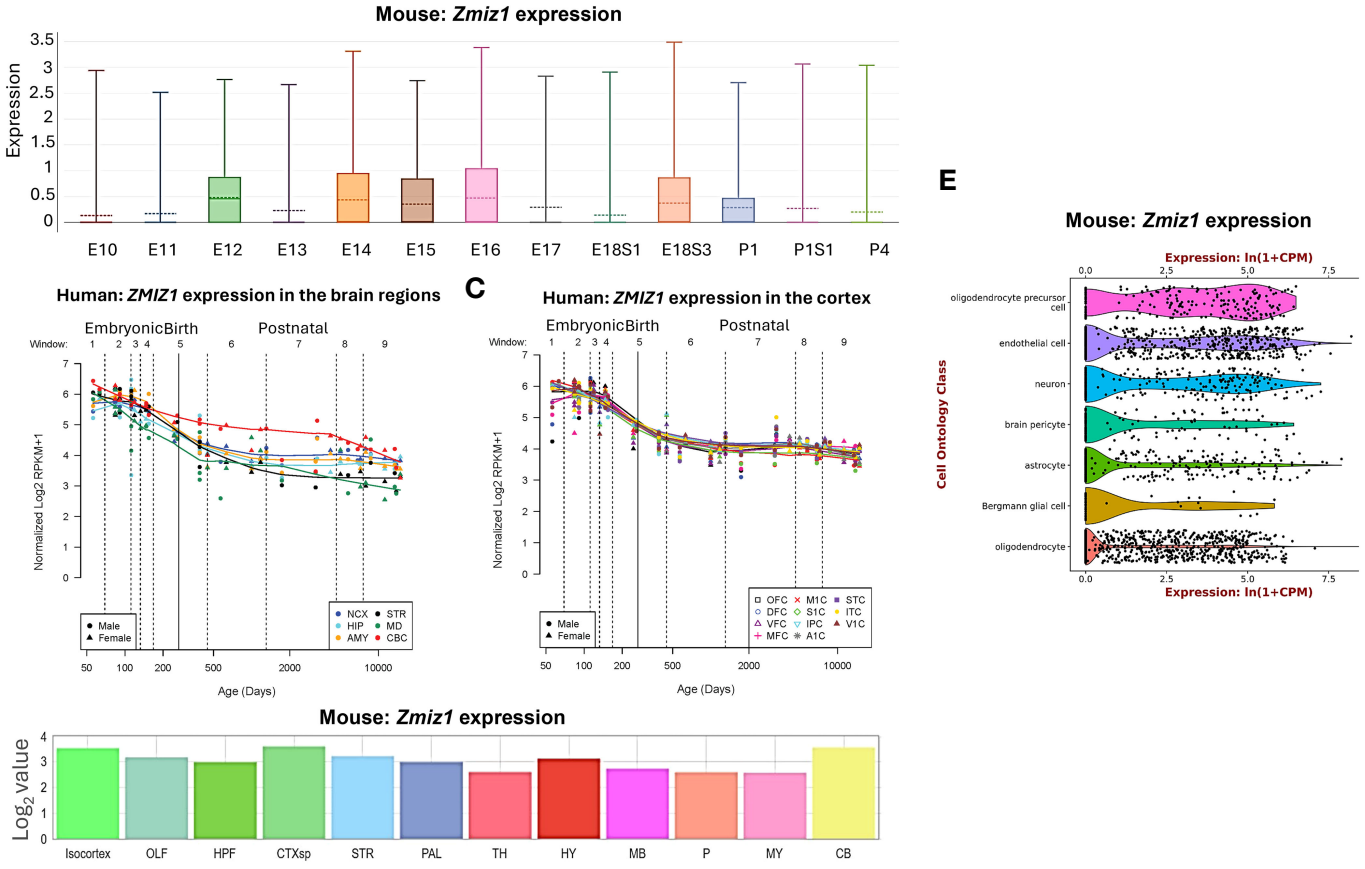
*Zmiz1* expression in the brain. **(A)** *Zmiz1* expression during mouse cortical development from embryonic day **(E)** 10.5 to postnatal day (P) 4 (adapted from single cell dataset, Di Bella, et al., 2021 (13). https://doi.org/10.1038/s41586-021-03670-5). **(B, C)** *Zmiz1* expression during human brain development across brain regions **(B)** and across cortical regions **(C)**. Window (W) 1-4: Embryonic and early to mid-fetal, 5: Birth, 6-9: Later postnatal. NCX-neocortex, HIP-hippocampus, AMY- amygdala, STR-striatum, MD-mediodorsal nucleus of thalamus, CBC-cerebellar cortex, MFC-medial prefrontal cortex, OFC-orbital prefrontal cortex, DFC-dorsolateral prefrontal cortex, VFC-ventrolateral prefrontal cortex, M1C-primary motor cortex, S1C-somatosensory cortex, IPC-posterior inferior parietal cortex, A1C-primary auditory cortex, STC-superior temporal cortex, ITC-inferior temporal cortex, V1C-primary visual cortex. Adapted from PsychENCODE (14), Human brain development, Sestan lab, http://development.psychencode.org/#. **(D)** Murine *Zmiz1* expression in different brain regions; isocortex, olfactory areas (OLF), hippocampal formation (HPF), cortical subplate (CTXsp), striatum (STR), pallidum (PAL), thalamus (TH), hypothalamus (HY), midbrain (MB), pons (P), medulla (MY), and cerebellum (CB) (adapted from Allen Brain Atlas). **(E)** *Zmiz1* expression in different cell types in the brain; oligodendrocytes precursor cell, endothelial cell, neuron, brain pericyte, astrocyte, Bergmann glial cell and oligodendrocyte (https://tabula-muris.ds.czbiohub.org/) (15).

*Zmiz1* expression levels vary depending on the developmental stage and tissue type, including brain, thyroid, ovary, retina, and lungs (11, 12). In developing mice, *Zmiz1* expression is detected on embryonic day (E) 8.5 in the developing neural tube, somites, and mesoderm. At later embryonic stages, *Zmiz1* expression is observed in various organs and tissues, including the brain, lung, liver, heart, and kidney (12). To understand Zmiz1*’s* role in brain development, we first investigated its temporal expression profile in the brain. In the developing murine brain, *Zmiz1* expression peaks during embryonic stages, from E12 to E18, followed by a decline in early and late postnatal stages (P) 1-4, suggesting a potential involvement in early neurodevelopmental events such as neurogenesis (**Figure 1A**). This is consistent with the expression pattern in the developing human brain, where *Zmiz1* expression is highest during embryonic development and decreases postnatally (14) (**Figures 1B, C**).

Spatially, *Zmiz1* is expressed throughout the brain, however, its expression is the highest in the cortex and cerebellum (**Figures 1B–D**). *Zmiz1* is also moderately expressed in olfactory areas, hippocampal formation, cortical subplate, striatum, pallidum, thalamus, hypothalamus, midbrain, pons, and medulla. *Zmiz1* expression is observed throughout different cortical regions, such as olfactory, visual, somatosensory, auditory, and motor regions (**Figure 1C**). Analysis of single-cell datasets from mouse brain reveals *Zmiz1* expression in a multitude of cell types, including neurons, endothelial cells, oligodendrocyte precursor cells, brain pericyte, astrocyte, Bergmann glial cells, and oligodendrocytes (15) (**Figure 1E**). Furthermore, the Allen Brain single-cell database (https://portal.brain-map.org/atlases-and-data/rnaseq), specifically the Whole Cortex & Hippocampus – 10X genomics (2020) and 10X-smart-seq taxonomy (2021) datasets, suggests *Zmiz1* expression in both glutamatergic and GABAergic neurons. However, its expression is stronger in excitatory neurons compared to GABAergic neurons (16). Interestingly, a previous study (17) described expression of *Zmiz1* in the cortex of embryonic and postnatal mice and reported expression in the cortical ventricular/subventricular zones, which contain the progenitors that give rise to cortical excitatory neurons.

**Figure 2:**
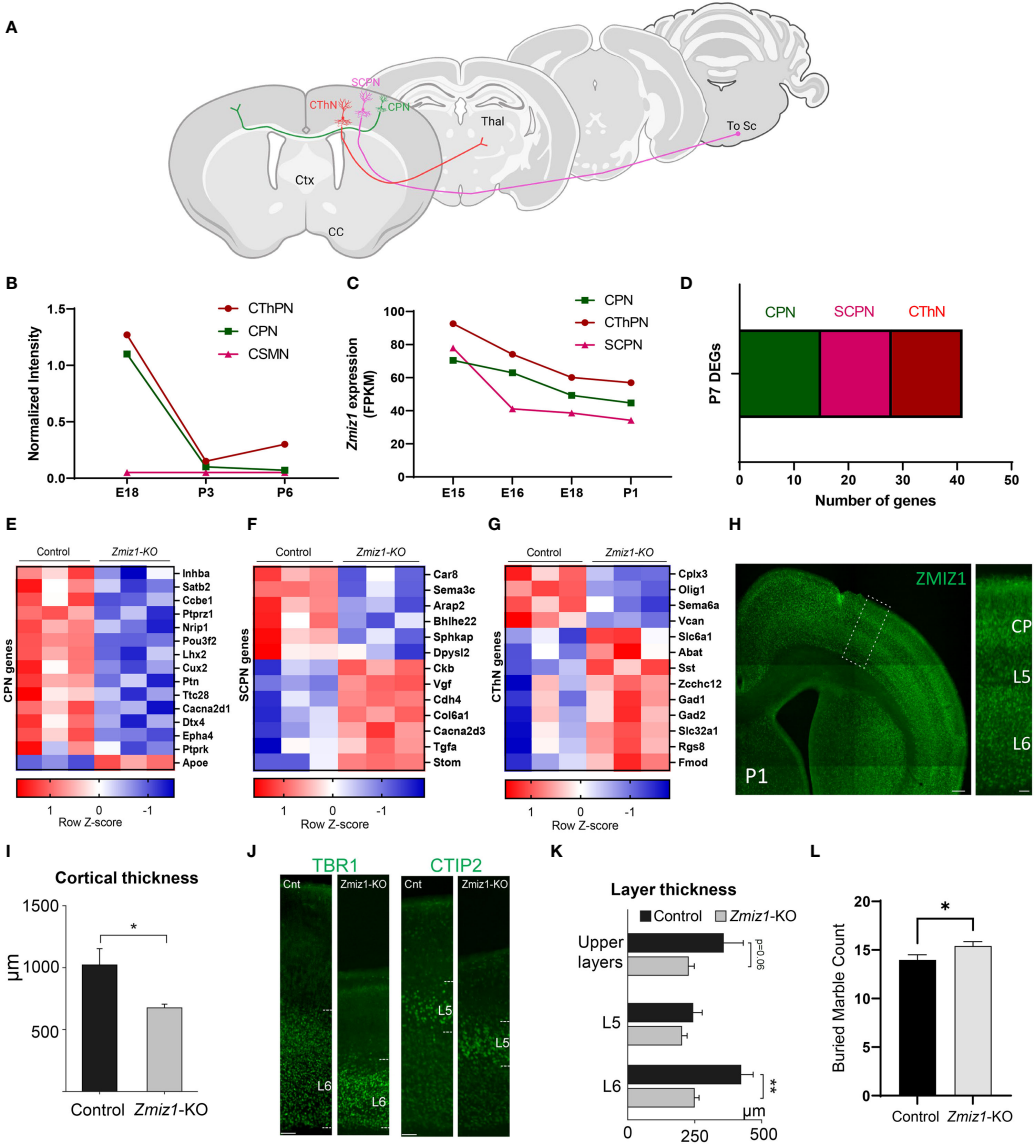
**(A)** Schematics of the main classes of excitatory projection neuron subtypes analyzed: Callosal projection neurons (CPN), Subcerebral projection neurons (SCPN), and Corticothalamic projection neurons (CThPN). **(B)** *Zmiz1* expression in cortical neuron subtypes CPN, SCPN, and CThPN at E18, P3, and P6. Adapted microarray data from Galazo et al., 2016 (17). **(C)** *Zmiz1* expression CPN, SCPN, and CThPN at E15, E16, E18, and P1. Adapted data from DeCoN (18). **(D)** Stacked plot of overlapping P7 DEGs Zmiz1-KO vs Control RNA-seq from this study with CPN, SCPN, and CThPN genes from DECON. E-G) Heatmap plot for CPN **(E)**, SCPN **(F)**, and CThPN **(G)** genes in the P7 DEGs list. RNA-seq performed in n=3 samples per genotype. **(H)** ZMIZ1 protein distribution shown by immunolabeling in P1 cortex. ZMIZ1 immunolabeling is strong in layer 6 and cortical plate (CP), which contains developing upper layer neurons at this time point. **(I)** Total cortical thickness analysis performed in motor cortex at P3 (n= 5 controls, n=3 *Zmiz1*-KO, unpaired t-test, *p<0.05). **(J)** Cortical layer staining with TBR1 for layer 6 and CTIP2 for layer 5 at P3 (n= 5 controls, n=3 Zmiz1-KO). **(K)** Layer thickness analysis performed at P3 (n= 5 controls, n=3 Zmiz1-KO, Anova, **p<0.01). **(L)** Marble burying behavioral test in adult mice (n= 32 controls, n=29 *Zmiz1*-KO, unpaired t-test, *p<0.05). Error bars, SEM. Scale bars, **(H)** low magnification panel 200 μm, **(H)** higher magnification cortical column 50 μm, **(J)** 100 μm.

To further investigate *Zmiz1* expression in excitatory neurons in the cortex, we used two datasets from studies (17, 18) investigating developmental transcriptome changes in three main classes of excitatory cortical projection neurons in mice: intracortical callosal projection neurons (CPN) located in the layer 2-3, subcerebral projection neurons (SCPN) in layer 5, and corticothalamic projection neurons (CThPN) in layer 6 (**Figure 2A**). Our analysis of these datasets revealed that *Zmiz1* is strongly expressed embryonically, but its expression rapidly decreases at neonatal stages, and it is highly reduced by P3 in these projection neuron subtypes (**Figures 2B, C**). Though *Zmiz1* is expressed across all three neuron subtypes, its expression is enriched in CThPN and CPN during embryonic development (**Figures 2B, C**). Interestingly, these neuron subtypes are importantly affected in several NDDs (19).

Collectively, these results indicate that Zmiz1 is broadly expressed across brain regions and cell types during embryonic development, but its expression is enriched in brain regions (cortex, hippocampus, and cerebellum) and cell types (CPN and CThPN), prominently impacted in ID, ASD, and other NDDs. These results underscored the need for further investigation of the function of Zmiz1 in the development of specific brain areas and cell types.

### *De novo* pathogenic variants tend to cluster in intrinsically disorganized regions of ZMIZ1 protein

**Figure 3:**
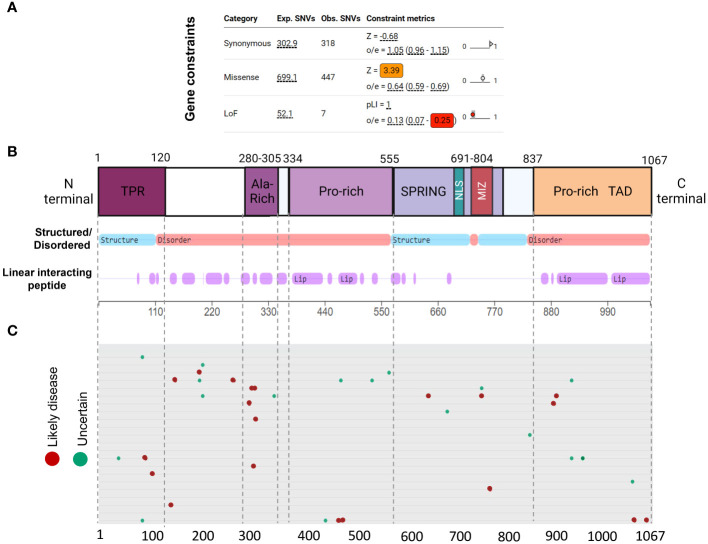
ZMIZ1 protein domains with associated mutations. **(A)** *ZMIZ1* gene constraints on synonymous, missense, and LoF mutations (adapted from SFARI). **(B)** ZMIZ1 protein structural features illustrating functional domains and intrinsically disordered regions (IDR) (adapted from mobidb.org) (20). **(C)** *Zmiz1* associated point mutations (adopted from UniProt Variants Viewer, www.uniprot.org/uniprotkb/Q9ULJ6/variants-viewer). Red - likely disease causing, green – likely benign, and cyan – uncertain.

Genetic constraint and protein structure analyses are crucial to understanding protein function and impact of gene mutations. Gene constraint measures the tolerance of a gene to a class of variation (e.g. loss-of-function) and serves as a key indicator of negative selection of mutations on a gene. Using SFARI’s methodology (oe-score; ratio of observed single nucleotide variants (SNVs) to expected SNVs), we determined *Zmiz1* gene constraint oe-scores for synonymous, missense, and loss-of-function mutations (**Figure 3A**). Lower oe-score values signify greater intolerance. *Zmiz1* showed an oe-score of 1.05 for synonymous mutations (observed SNVs 318/expected SNVs 302.9), and an oe-score of 0.64 for missense mutations (observed SNVs 447/expected SNVs 699.1). Importantly, *Zmiz1* has an oe-score of 0.13 for loss-of-function mutations (observed SNVs 7/expected SNVs 52.1), which indicates that only 13% of the expected loss-of-function variants were observed in sampled data. These results indicate a high constraint and high selective pressure on *Zmiz1*.

Intrinsically disordered proteins (IDPs) are known to facilitate the formation of protein-protein complexes, protein-DNA interactions, and the formation of membrane-less organelles via liquid-liquid phase separation. Because of these properties, IDPs have been proposed as key players in chromatin remodeling, transcriptional regulation, and formation of super-enhancers. This is consistent with reported functions of ZMIZ1 as a non-DNA binding transcriptional co-factor (21). We investigated ZMIZ1 protein structure to better understand its function. Our analysis indicates that, structurally, ZMIZ1 is an intrinsically disordered protein (**Figure 3B**). While 22.3% of ZMIZ1 encompasses structured functional domains, 66.40% corresponds to highly disordered regions (IDRs) (**Figure 3B**), therefore, ZMIZ1 qualifies as an IDP. The presence of Intrinsically disordered regions (IDRs) confers extremely dynamic behavior to proteins, which is essential to IDPs functions in multi-molecular complex formation. Specific subregions within IDRs known as Linear Interacting Peptides (LIPs) undergo disorder-to-order transitions when interacting with protein domains or nucleic acids, and are vital for phase transition (22, 23). Our analysis indicates that 50.30% of the ZMIZ1 protein structure is composed of LIPs (**Figure 3B**). The high content of IDRs and LIPs in the ZMIZ1 sequence suggests a functionally important role in transcriptional regulation, and potentially a novel role in the formation of multi-molecular complexes and super-enhancers via phase separation.

The ZMIZ1 exhibits a notable presence of *de novo* mutations (Uniprot variants viewer: https://www.uniprot.org/) (**Figure 3C**). Although Likely Disease-Causing *de novo* mutations and Uncertain nature *de novo* mutations are distributed throughout the ZMIZ1 protein sequence, they significantly clustered into four specific domains: the TPR, Alanine-rich, Central Proline-rich, and C-terminal Proline-rich domains (**Figure 3C**). Sixty-five percent of Disease-causing SNV mutations and 60.5% of Uncertain nature SNVs are in these domains. The Alanine-rich, Central Proline-rich, and C-terminal Proline-rich domains correspond to IDRs. When normalizing the mutation load by the length of these domains (percentage of mutations/number of amino acids), the Alanine-Rich stands out as the domain in ZMIZ1 accumulating the highest burden of Disease-causing mutations per amino acid (Alanine-rich 20%, TPR 1.66%, Central Proline-rich 0.9%, C-terminal Proline-rich 1.74%). The entire Alanine-Rich domain contains repeats of LIP sequences. LIPs are also highly prevalent in the Central Proline-rich, and C-terminal Proline-rich domains (**Figure 3B**).

Together, the high intolerance of Zmiz1 to mutation and its complex and highly disorganized protein structure suggests important functions in transcription regulation, and perhaps novel functions in the formation of multi-molecular complexes and super-enhancers.

### Zmiz1 is associated with recruitment of activating histone marks

ZMIZ1 is known to interact with subunits of the SWI/SNF complex and to bind transcription factors as a co-factor. However, the molecular mechanisms and interactions determining ZMIZ1 activity in transcriptional regulation and chromatin remodeling are far from understood. ZMIZ1, also called PIAS-Like Protein Zimp10, is a member of the PIAS family of proteins. PIAS are negative regulators of STAT signaling and are mainly known as transcriptional repressors, although they also have been shown to act as activators (24). Functions of ZMIZ1 as a co-activator of p53 and Androgen receptor-mediated transcription have been described (25, 26). Thus, whether ZMIZ1 functions as a co-repressor, co-activator, or both is not established.

**Figure 4:**
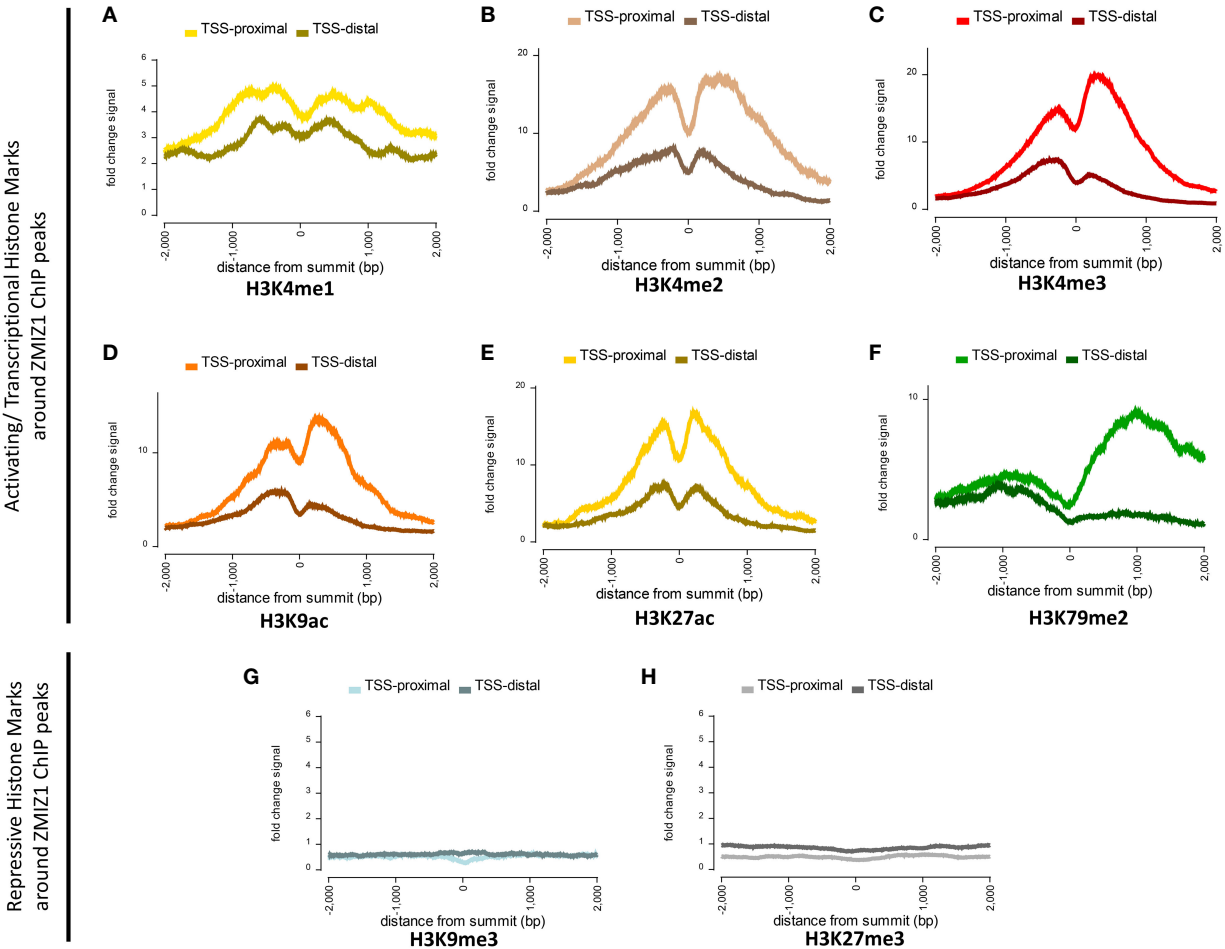
Highly enriched activating histone modifications mark around ZMIZ1 bound ChIP peaks in K562 human cell line. **(A–F)** Strong activating/transcriptional histone marks around ZMIZ1 bound ChIP peaks near TSS-proximal site: **(A)** H3K4me1, **(B)** H3K4me2, **(C)** H3K4me3, **(D)** H3K9ac, **(E)** H3K27ac, **(F)** H3K79me2. **(G, H)** No repressive histone marks around ZMIZ1 bound ChIP peaks: **(G)** H3K9me3, **(H)** H3k27me3. Adapted from Factorbook (27, 28), epigenetic profile, factorbook.org.

To investigate this question, we used Factorbook database (a web-based repository of integrative analysis associated with ENCODE ChIP-seq data) to analyze the presence of histone modification marks around ZMIZ1 chromatin binding sites (ZMIZ1-ChIP peaks) in K562 human cell line. We found a robust enrichment of activating histone marks, including H3K4me1, H3K4me2, H3K4me3, H3K9ac, H3K27ac, and H3K79me2, suggesting strong transcriptional activation of genes bound by ZMIZ1 peaks (**Figures 4A–F**) (27, 28). Conversely, repressive histone marks, such as H3K9me3 and H3K27me3, are minimally present around ZMIZ1-ChIP peaks (**Figures 4G, H**).

Histone activating marks stimulate transcription by promoting transcription initiation (29–31). Specifically, high enrichment of H3K27ac is typically associated with highly active loci and has been used to determine the presence of enhancers and super-enhancers (32, 33). The strong enrichment of activating histone marks associated with ZMIZ1 bound peaks, together with its disorganized structure and high content of LIPs, indicate that ZMIZ1 primarily functions as a co-activator and are consistent with the predicted functions in the formation of multi-molecular complex and super-enhancers.

### Loss of Zmiz1 disrupts expression of cortical neuron development genes and affects cortical development

**Figure 5:**
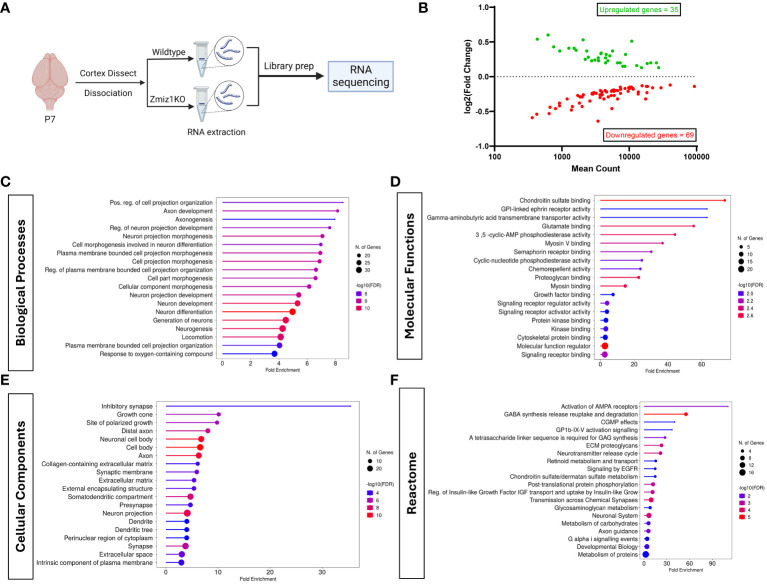
Zmiz1 regulates neuronal developmental processes. **(A)** Schematic diagram depicting RNA sequencing strategy. **(B)** MA plot of differentially expressed genes between wildtype and *Zmiz1*-KO cortex. **(C–F)** Top Gene Ontology (GO) analysis on differentially expressed genes for biological processes **(C)**, molecular functions **(D)**, cellular component **(E)**, and Reactome **(F)** (FDR ≤ 0.05).

To begin to understand the role of Zmiz1 in cortical development, we investigated the effect of its loss of function in the mouse cortex. We conditionally deleted *Zmiz1* in the cortical progenitors, using an *Emx1-*Cre mouse line (*Zmiz1*-KO), and assessed transcriptomic changes at P7 (**Figure 5A**). We found 104 differentially expressed genes (DEGs) in the *Zmiz1*-KO cortex, out of which 35 were downregulated genes and 69 were upregulated genes (**Figure 5B**). We performed Gene Ontology (GO) analysis on DEGs to evaluate the biological and cellular processes affected. Biological processes such as neurogenesis, neuron development and differentiation, axon development, and neuron projection morphogenesis were significantly affected in the *Zmiz1*-KO cortex (**Figure 5C**). Molecular functions affected included ephrin signaling, glutamate signaling, and semaphorin signaling (**Figure 5D**). Specific cellular components associated with DEGs include synapses, axons, dendrites, and growth cones (**Figure 5E**). Reactome pathways such as AMPA receptor activation, neurotransmitter release cycle, chemical synapse transmission, and GABA signaling (**Figure 5F**) were significantly affected in *Zmiz1*-KO cortex.

Next, we assessed transcriptome changes in the main classes of cortical projection neuron subtypes CPN, SCPN, and CThPN (**Figure 2A**). We conducted a correlation analysis of P7 *Zmiz1*-KO cortex DEGs in our study with gene clusters associated with CPN, SCPN, and CThPN from the DeCoN dataset (18). According to DeCoN classification, 15 of the *Zmiz1*-KO cortex DEGs are cataloged as CPN genes, 12 DEGs are SCPN genes, and 6 are CThPN genes (**Figure 2D**). Noteworthy CPN-DEGs encompass Inhba, Satb2, Ccbe1, Ptprz1, Nrip1, Pou3f2, Lhx2, Cux2, Ptn, Ttc28, Cacna2d1, Dtx4, Epha4, Ptprk, and Apoe. SCPN-DEGs consist of Car8, Sema3c, Arap2, Bhlhe22, Sphkap, Dpysl2, Ckb, Vgf, Cdh4, Col6a1, Cacna2d3, and Stom, while CThPN-DEGs include Cplx3, Sema6a, Vcan, Zcchc12, Rgs8, and Fmod (**Figures 2E–G**). The observed distributed gene expression profile across cortical neuron subtypes suggests that the loss of Zmiz1 does not selectively impact specific neuron subtypes, although it might have a stronger impact on CPN development.

We performed immunolabeling to detect ZMIZ1 protein in the cortex of P1 mice and detect expression in all cortical layers. We found that ZMIZ1 protein expression is strong in layer 6, where CThPN reside in the cortex, and in the developing upper layers of the cortical plate that will differentiate into layer 2-3 and contain most CPN (**Figure 2H**). However, ZMIZ1 expression appears weaker in layer 5 compared to layer 6 and developing upper layers (**Figure 2H**). These results are consistent with the enriched *Zmiz1* mRNA expression in CThPN and CPN previously reported by neuron subtype-specific transcriptome analysis (17, 18).

ZMIZ1 protein cortical expression pattern suggests that *Zmiz1* mutation may affect the development of multiple cortical neuron types, but perhaps it preferentially affects CThPN and CPN subtypes. To investigate this question, we performed a gross morphological analysis of the cortex. We first measure the overall cortical thickness from pia to white matter border in *Zmiz1-KO* and control mice motor cortex at P3. We found a significant decrease in motor cortical thickness in *Zmiz1-KO* mice (**Figure 2I**). Next, we analyzed the thickness of layers 6, 5, and developing Upper layers. To measure layer 6 thickness, we performed immunolabeling for layer 6 and CThPN marker TBR1 (34, 35) and measured the distance from the bottom border to the top of the labeled layer (**Figure 2J**). Similarly, we immunolabeled for layer 5/SCPN marker CTIP2 and measured layer 5 thickness (36, 37). The thickness of developing upper layers were measured from the top border of the CTIP2+ layer 5 to the pia (**Figure 2J**). We found a significant reduction in layer 6 thickness in *Zmiz1-KO* mice compared to controls at P3 (**Figure 2K**). We also found a reduction of in the thickness of the developing upper layers in *Zmiz1-KO* mice, although this result did not reach statistical significance (p=0.06), possibly because not all neurons that will eventually populate the upper layers have completed their migration and settled in the upper layers at P3. We found no statistically significant difference or trend toward reduction in layer 5 thickness (**Figure 2K**). These changes in motor cortex morphology may lead to alteration of cortical function. Given that Zmiz1 mutation has recently been associated with various NDDs, including ASD, we performed a behavioral assessment using the marble burying task, which is used in ASD rodent models to assess repetitive/compulsive behaviors typically observed in ASD (38). We found an increase in motor repetitive behaviors in adult *Zmiz1-KO* mice compared to controls (**Figure 2L**).

Together, our ZMIZ1 protein expression data and the cortical and behavioral alterations of *Zmiz1-KO* mice strongly suggests that *Zmiz1* mutation affects cortical function and may underlie some of the neurodevelopmental phenotypes observed in humans carrying Zmiz1 mutations.

### Zmiz1 is an ASD risk gene

Zmiz1 plays a pivotal role in various biological processes, spanning embryonic development, angiogenesis, immune response, and has been associated with conditions such as cancer, leukemia, and diabetes (11, 39–46). In recent years, the link between Zmiz1 mutations and NDDs, particularly ASD, has emerged (9, 10, 47–49). The Simons Foundation Autism Research Initiative (SFARI) (https://www.sfari.org/) and the Online Mendelian Inheritance in Man (OMIM) (https://www.omim.org/) database establish connections between Zmiz1 and clinical neurologic disorders, including intellectual disability (ID), attention deficit hyperactivity disorder (ADHD), ASD, and aggression. SFARI categorized *Zmiz1* as a strong candidate for syndromic ASD with the presence of 24 rare *de novo* pathogenic variants, but no studies have explored yet the potential contribution of *Zmiz1* in ASD.

**Figure 6:**
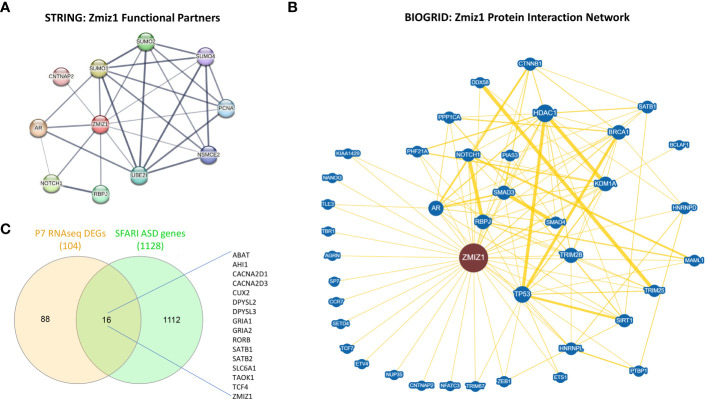
ZMIZ1 associated likely proteins interactome network. **(A)** ZMIZ1 interacting functional protein association network from STRING database (string-db.org) (50). **(B)** ZMIZ1 curated protein-protein interaction network based on an interaction database repository of physical, chemical, and genetic evidence, adapted from BioGRID database (thebiogrid.org) (51). Greater node size represents increased connectivity and thicker edge sizes represent increased evidence supporting the evidence. Well studied ZMIZ1 interactors include NOTCH1, TP53, AR, and Smad3/4. **(C)** Venn diagram depicting P7 RNAseq DEGs overlap with SFARI autism risk gene module.

Since *ZMIZ1* encodes a transcriptional co-regulator, we investigated the correlation between ZMIZ1 interacting partners and ASD risk genes. Using STRING database (50) and BioGrid database (51), we unveiled a network of functional interactions, indicating potential direct and indirect connections. As expected, well-known ZMIZ1 interactors such as TP53, Notch1, SMAD3/4, and AR are present in the interacting network, confirming the validity of the analysis. Also, novel potential interactors such as PCNA, CNTNAP2, SUMO1, SUMO2, SUMO4, UBE2I, RBPJ, NSMCE2, CTNNB1, NANOG, SATB1, SETD4, TBR1, BRCA1, HDAC1, NFATC3, HNRNPD, and ETS1 emerged in the ZMIZ1 network (**Figures 6A, B**). Cross-referencing these interactors with the SFARI gene scoring database reveals several ZMIZ1-interacting ASD risk genes including CTNNB1, CNTNAP2, SATB1, TBR1, HNRNPD, and ETS1, further underscoring the potential role of Zmiz1 in ASD pathogenesis.

Additionally, we compared the DEGs obtained in our RNAseq experiments from *Zmiz1*-KO cortex at P7 with SFARI Autism gene modules and identified that 16 out of 104 DEGs uniquely map to SFARI Autism risk genes. These genes, including *ABAT, AHI1, CACNA2D1, CACNA2D3, CUX2, DPYSL2, DPYSL3, GRIA1, GRIA2, RORB, SATB1, SATB2, SLC6A1, TAOK1, TCF4*, and *ZMIZ1*, signify a potential regulatory role of ZMIZ1 in ASD risk gene expression in the developing cortex (**Figure 6C**). Network interactors, particularly ZMIZ1-interacting ASD risk genes and downstream Zmiz1-regulated ASD risk genes shed light on the intricate network governing Zmiz1 functions in neurodevelopment, particularly in the context of ASD, emphasizing the need for further investigations.

### Zmiz1 is abundantly expressed in the dendritic-axonal compartment

**Figure 7:**
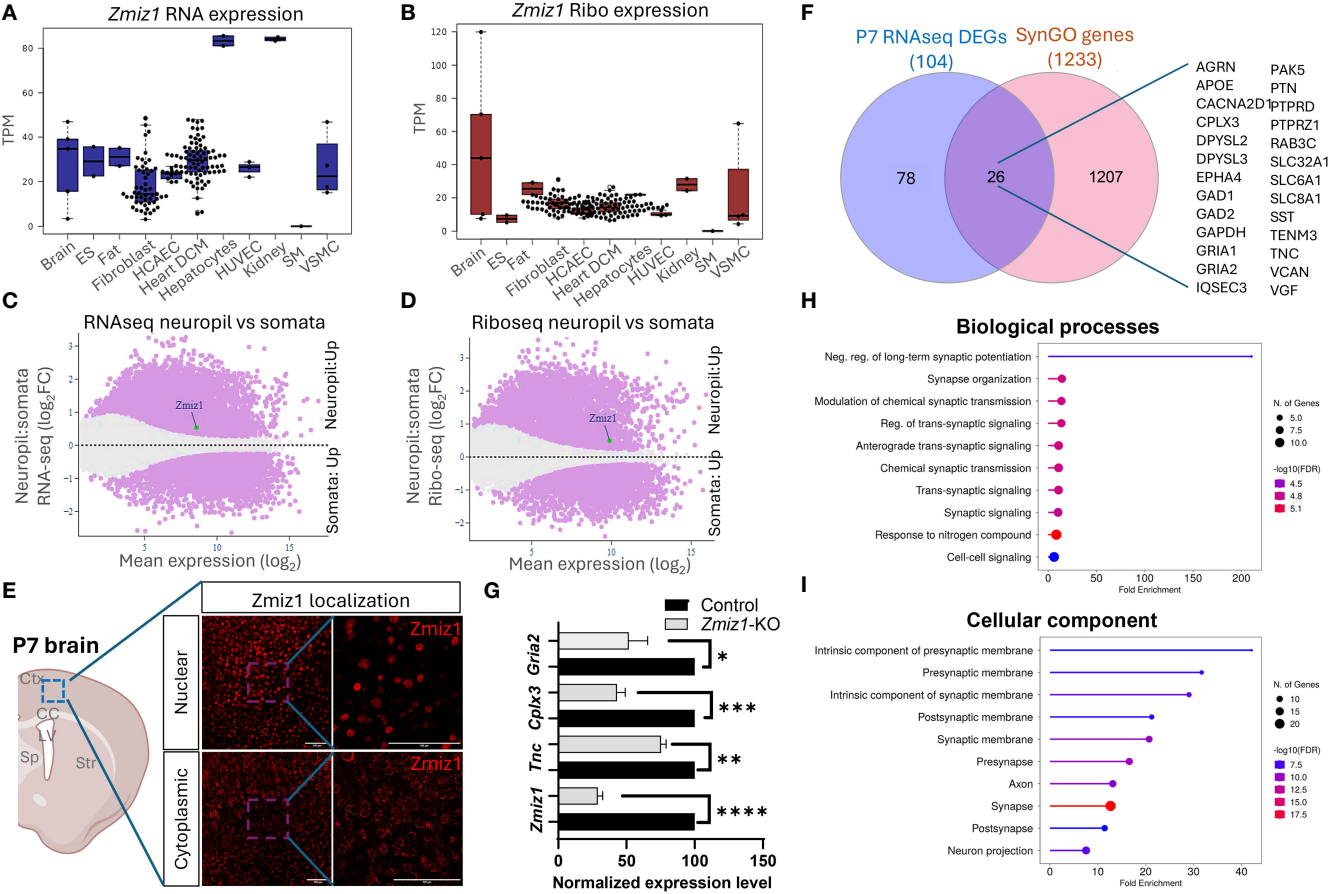
*Zmiz1* mRNA expression and translation in the neuronal somato-dendritic compartment. **(A, B)** *Zmiz1* transcription (RNA-seq) **(A)** and translation (Ribo-seq) **(B)** in different tissue and cell types including brain (high), embryonic stem cells (ES), fat, fibroblast, coronary artery endothelial cells (HCAEC), heart dilated cardiomyopathy (DCM), hepatocytes, human umbilical vein endothelial cells (HUVEC), kidney, skeletal muscle (SM), and vascular smooth muscle cells (VSMC). Adapted from Chothani et al., 2022 (52) (https://smorfs.ddnetbio.com). **(C, D)** Differential *Zmiz1* transcripts in neuropils compared to cell body from RNA-seq **(C)** and Ribo-seq **(D)**. Neuropils: dendrites and axon compartment. Adapted from Glock et al., 2021 (53). (https://public.brain.mpg.de/dashapps/localseq/). Purple – Significant (adjusted p-value < 0.05) and gray - not significant (adjusted p-value ≥ 0.05). **(E)** ZMIZ1 protein nuclear and cytoplasmic localization in murine cortical neurons. Ctx - cortex, CC – corpus callosum, LV – lateral ventricle, Sp – septum, and Str – striatum. **(F)** Venn diagram depicting P7 RNAseq overlap with SynGO genes. **(G)** qPCR analysis confirms downregulation of *Zmiz1* and genes involved in synaptic transmission (*Gria2, Cplx3, Tnc*) in *Zmiz1*-KO samples (light grey) compared to controls (black) (n = 3 per genotype, one-way Anova, *p<0.05, **p<0.01) **(H, I)** Plots for SynGO ontology on overlapped 26 genes from **(F)** for biological processes **(H)** and cellular components **(I)**.

The dynamic nature of mRNA and protein regulation renders mRNA expression alone insufficient to predict protein levels. To better understand the relationship between *Zmiz1* mRNA and ZMIZ1 protein expression, we used the atlas of mRNA translation which utilizes RNAseq and Riboseq (52). Interestingly, *Zmiz1* mRNA is strongly expressed in the brain and other tissues. However, *Zmiz1* mRNA translation is stronger in the brain compared to other tissues and cell types, suggesting specific functions and regulatory significance in brain development (**Figures 7A, B**).

Transcriptomic analysis reveals the involvement of Zmiz1 in a variety of neurodevelopmental processes, including neurogenesis, neuron differentiation, and synaptic signaling, which span a wide temporal window and affect different cell types. The above-described structural characteristics of ZMIZ1 protein allow for interaction with a variety of binding partners, which may contribute to the diversity of Zmiz1 regulatory functions. In addition, multiple Zmiz1 isoforms can contribute to this diversity. To deepen our understanding of Zmiz1 functions relative to its expression, we delved into the mRNA translation landscape in the synaptic neuropil dataset (53), surveying mRNA localized and translated in distinct neuronal compartments: cell body/somata and dendrites/axon. Thousands of transcripts displayed differential transcription and translation between somatic and neuropil compartments. *Zmiz1* emerged as one of the 800 genes showing significantly higher transcription and translation in the neuropil compared to soma: RNAseq (*Zmiz1* Mean expression (log2): 8.586/Neuropil : Somata Fold change (log2): 0.532) and Riboseq (*Zmiz1* Mean expression (log2): 9.888, Neuropil : Somata Fold change (log2): 0.494) (**Figures 7C, D**) indicating its distinct expression profile in these compartments. Importantly, immunolabeling in P7 mouse cortices with antibodies detecting either C-terminal or N-terminal epitopes in ZMIZ1 revealed protein localized either in the nucleus (C-terminal), or the cytoplasm/neuropil (N-terminal) (**Figure 7E**). While the presence of ZMIZ1 in the nucleus is consistent with its known function as a transcriptional regulator, presence of ZMIZ1 in neuropil/cytoplasm suggests additional and novel roles, perhaps related to the synaptic signaling processes emerging in our transcriptome analysis.

We further explore whether Zmiz1 regulates synaptic function and synaptic gene expression. For that, we utilized the SynGO database (54). We correlated and interpreted DEGs in the P7 *Zmiz1*-KO cortex as a function of synapse biology using SynGO annotations and enriched ontologies. Among the 104 DEGs, 26 were mapped to unique SynGO annotated genes (**Figure 7F**). Noteworthy, genes include *Gria1, Gria2, Cacna2d1, Tnc, Sst, Gad1, Gad2, Epha4, Apoe, Dpsyl2, Slc8a1, Agrn, Cplx3, Dpsyl3, Gapdh, Iqsec3, Pak5, Ptn, Ptprd, Ptprz1, Rab3c, Slc32a1, Slc6a1, Tenm3, Vcan, and Vgf*. Biological processes such as synapse organization, synaptic signaling, synaptic transmission, and cellular components such as presynaptic membrane, postsynaptic membrane, pre-synapse, axon, and post-synapse are significantly enriched (**Figures 7G, H**). We performed qPCR to confirm changes in expression of a subset of these DEGs encoding components of presynaptic, postsynaptic, extracellular synaptic elements (Gria2, Tnc, and Cplx3) and confirmed their downregulation in *Zmiz1-KO* compared to controls mice (**Figure 7G**). Overall, our findings revealed that ZMIZ1 protein is expressed in neuropil/cytoplasm as well as in the soma/nucleus, which underscores the multifaceted role of Zmiz1 in cortical development and emphasizes its potential impact on neuronal connectivity and synaptic function.

## Discussion

Neurodevelopmental disorders, including ID, ASD, ADHD, communication disorders, motor disorders (including tic disorders), and learning disorders per the Diagnostic and Statistical Manual of Mental Disorders 5 (DSM-5), exhibit a multifaceted genetic basis (55). *De novo* mutations significantly contribute to NDD etiology, with individuals with ASD displaying a higher burden of *de novo* loss-of-function mutations, particularly in genes highly expressed during brain development (6, 7). Notably, these mutations are prevalent in genes associated with chromatin remodeling and histone modification, and individuals with more severe developmental disorders bear a higher burden of *de novo* mutations. Our examination of Zmiz1 reveals its expression patterns and structural disorderliness as crucial indicators of its physiological functions. Transcriptomic analysis highlights dysregulation in gene expression profiles related to neuron differentiation. Furthermore, Zmiz1 mutations, its downstream transcriptional targets, and protein interactors collectively constitute a combinatorial risk factor for ASD. Our study reveals the presence of *Zmiz1* in the axon-dendritic-somatic compartment, indicating novel functions of Zmiz1 beyond transcriptional regulation. In terms of gene regulation and chromatin remodeling, Zmiz1 function is mediated by activating histone modifications, adding complexity to the genetic network implicated in NDDs (7, 56, 57).

The ZMIZ1 protein domains play critical roles in its function, including DNA binding and protein-protein interactions. Low complexity structural features, IDRs and LIPs, are increasingly recognized for their functional role in protein-protein/protein-nucleic acids interactions, signaling pathways, and transcriptional control (22, 23). The presence of IDRs and LIPs in ZMIZ1 is consistent with its roles in the formation of multi-molecular complexes and transcriptional control, and potentially in the formation of super-enhancers. Recently, *Zmiz1* has been identified as a super-enhancer associated gene, important for regulation of super-enhancer activity in response to estrogen during uterine development (58). Investigating the specific sequence determinants of these regions in the ZMIZ1 protein and their functional impact could offer valuable insights into the mechanisms governing Zmiz1-mediated signaling and regulation.

Zmiz1-associated neuropsychiatric pathologies due to mutational load as such *de novo* mutation/missense variant/translocation/chromosomal rearrangements lead to complex behavioral disorders including anxiety, social communication, speech delay, memory recall, ID, and developmental delay. Beyond NDDs, Zmiz1 is associated with depression (59), multiple sclerosis (MS) (60), and Hirschsprung disease (HD) (61). Analysis of differentially expressed genes in the dentate gyrus of a depression mouse model indicates that Zmiz1 potentially functions in regulating key upregulated hub genes, linking it to the pathogenesis of depression (59). GWAS studies in MS patients identified downregulation of Zmiz1 in autoimmunity and as an MS risk gene (60). Furthermore, a case study revealed a *de novo* pathogenic variant in *ZMIZ1* in a patient with developmental delay and Hirschsprung Disease (HD), suggesting HD as part of the clinical spectrum of Zmiz1-associated NDDs and highlighting Zmiz1’s potential importance in the development of the enteric nervous system (61).

Cerebral cortex development and malformations are pivotal in the pathogenesis of NDD (62–64). Major excitatory neurons, including cortical projection neuron subtypes such as CPN, SCPN, and CThPN, are crucial for higher-order functions and sensory integration. CPN, particularly affected in Autism, exhibits high *Zmiz1* expression during embryonic development, suggesting a potential regulatory role in their generation and maturation (65) (**Figure 5B**). CPN play a fundamental role in inter-hemispheric connections through the corpus callosum, contributing to high-level associative tasks in social behavior and cognition (66, 67). Disruptions in CPN and corpus callosum development, a common feature in neurologic disorders like ASD and ID, lead to impaired emotional, social, communication, and cognitive functions (63, 68–72).

*ZMIZ1* mRNA is a target of Fragile X Mental Retardation Protein (FMRP), a well-studied protein in autism (73). Moreover, ZMIZ1 interacts with Brg1 (SMARCA4) and BAF57 (SMARCE1) proteins of the SWI/SNF-like BAF chromatin remodeling complex, crucial for neuronal differentiation, dendritic development, and synapse development (47, 74–77). Furthermore, Zmiz1 serves as a transcriptional co-activator for p53 (25) and Notch1 (40, 46), key regulators of dendritic development and subsequently cortical circuitry (78–80). Imbalances in excitation and inhibition, linked to distorted connectivity, are associated with ASD and various neurological disorders (81). These developmental and structural abnormalities manifest as behavioral disorders, including intellectual impairment, speech development delay, seizures, impaired motor function, social-behavioral deficits, and cognitive deficits which are observed phenotypes in human patients with *Zmiz1 de novo* mutations and correlate to ASD patients (3, 9, 10, 47–49, 82–87).

Interestingly, differential *Zmiz1* expression in the neuropil and cytoplasmic/membrane localization suggests an unknown axon-dendritic-somatic or synapse-related function of Zmiz1. This may occur through two potential scenarios: first, Zmiz1 acts as a transcriptional regulator influencing gene expression profiles in diverse developmental processes within these compartments; second, Zmiz1, beyond nuclear localization, has isoforms and variants in other regions like the membrane, axon, dendrites, and pre- and post-synaptic domains, warranting further investigation into its functional role.

### Future directions

**Figure 8:**
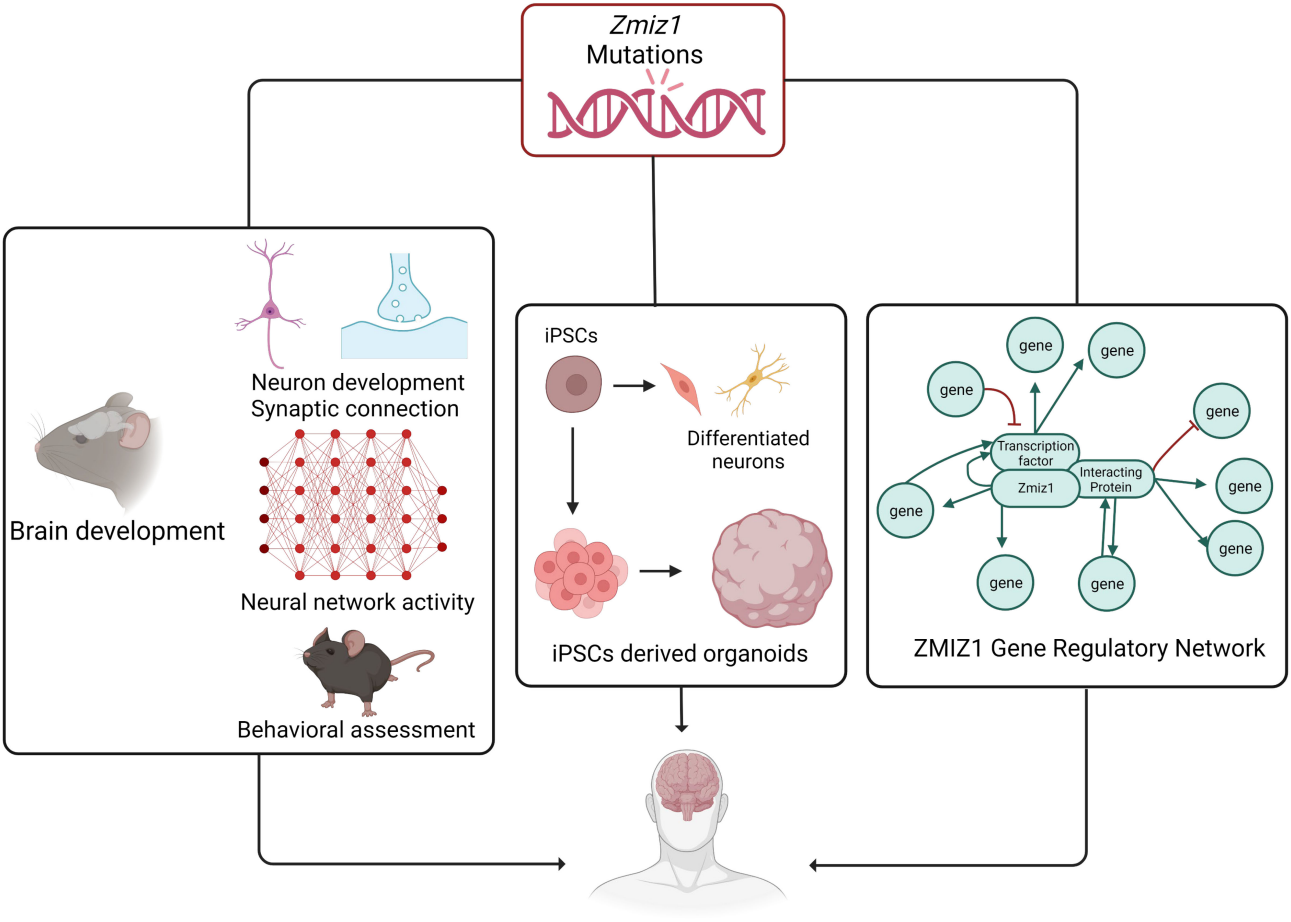
The systematic approach to elucidate the role and regulatory mechanism of Zmiz1 during brain development. The functions of numerous NDD risk genes have been assessed using a murine model and *in-vitro* approaches. Zmiz1 mutational outcome can be studied by understanding its function and evaluating its mutation disruptive actions. This can be studied using 1) murine model assessing its functional role in brain development which includes neuron development and synaptic connectivity, overall network level circuity activity, and ASD-related behavioral assessment, 2) iPSCs-derived models and organoids to better manipulate the experimental paradigm and assess its cellular functions, and 3) sequencing and molecular approaches to establish Zmiz1 gene regulatory network. This knowledge will be beneficial to understanding and finding therapeutic approaches targeting Zmiz1-associated syndromes and potentially targeting a broader range of NDD conditions.

The identification of *ZMIZ1* as an ASD risk gene offers valuable insights into the genetic mechanisms of ASD. Early diagnosis is crucial for intervention, and *ZMIZ1* mutation testing may aid in identifying ASD risk, enabling early diagnosis. The functional consequences of *ZMIZ1* mutations in ASD are unknown. Studying Zmiz1 functions in brain development can shed light on mechanisms underlying developmental defects and ASD due to Zmiz1 mutations. Integrated molecular, cellular, and functional studies in animal models and human iPSC/organoids will provide insights into the potential role of Zmiz1 in ASD pathogenesis (**Figure 8**). Zmiz1 emerges as a pivotal locus for investigating ASD and NDD etiology, offering valuable insights into disorder. Further research is essential to comprehensively understand Zmiz1 functions in brain development and how its mutations contribute to developmental disorders, potentially guiding therapeutic approaches and targets.

## Methods and materials

### Animals

All animal experiments were approved by Tulane University Institutional Animal Care and Use Committee (IACUC) and performed in accordance with institutional and federal regulations. *Zmiz1f/f* (88) mouse was generously donated by Dr. Mark Y. Chiang, University of Pennsylvania. *Emx1-Cre* (89) mouse (Stock No. 005628) was purchased from Jackson Laboratory.

### Immunohistochemistry

Mice were transcardially perfused with ice-cold PBS and then with 4% PFA. Brains were dissected and post-fixed with 4% PFA at 4°C overnight. The brain was sectioned coronally at 50 μm using a vibratome (Leica). Floating brain sections were then blocked using Cas block (Thermo Fisher, 008120) and incubated with primary antibodies overnight at 4°C followed by 1-hour secondary antibody incubation at room temperature. Sections were mounted using DAPI Fluoromount-G (SouthernBiotech, 0100-20). Antibodies used include ZMIZ1 (Thermo Fisher Scientific, PA5-50742, N-terminal ZMIZ1, 1:500) and ZMIZ1 (Cell Signaling Technology, 89500, C-terminal ZMIZ1, 1:500). Secondary antibody used, donkey anti-Rabbit 555 (Invitrogen, A31572, 1:1000) and donkey anti-mouse 488 (Invitrogen, A11001, 1:1000). Sample size, n = 3.

For cortical layer analysis, antibodies used include rabbit anti-Tbr1 (Abcam, ab31940, 1:500), rat anti-Ctip2 (Abcam, ab18465, 1:250), goat anti-rabbit 488 (Invitrogen A11008, 1:1000) and goat anti-rat 555 (Invitrogen A11006, 1:1000).

### RNA sequencing and gene expression analysis

P7 cortices were dissected (n = 3 per genotype). RNA extraction and processing for sequencing were performed as previously described (90, 91). Briefly, total RNA was extracted from wildtype and *Zmiz1-KO* cortex. RNA concentration and RNA integrity were determined using qubit (Thermo Fisher) and bioanalyzer (Agilent). RNA library was prepared, quantified, and verified using TruSeq RNA Library Prep Kit v2, Qubit dsDNA High Sensitivity Assay kit, and Bioanalyzer DNA1000 assay kit respectively. Verified samples were sequenced using either NextSeq 500/550 High Output Kit v2.5 (150 Cycles) (Illumina, 20024907). RNA-seq data analysis was performed using Illumina BaseSpace Sequence Hub. Briefly, sequenced reads were aligned to mouse (mm10) reference genome with the RNA-Seq alignment tool (STAR aligner), and differentially expressed genes (DEG) were determined using the RNA-Seq Differential Expression tool (DESeq2). Detailed workflow of DESeq2 and code can be found in (92). Gene ontology analysis of DEG was performed using ShinyGO 0.77 (bioinformatics.sdstate.edu/go/) (93). P7 cortex sequencing data have been deposited in the Gene Expression Omnibus (GEO) database with accession no. **GSE225435**.

### Quantitative PCR (qPCR)

To determine the mRNA expression levels, 1 μg of extracted RNA was transcribed into cDNA using the iScript Reverse Transcription Supermix (Bio-Rad, 1708840). qPCR was performed using the PerfeCTa SYBR Green SuperMix (Quantabio, 95071) on CFX96 system (Bio-Rad). Relative gene expression was determined using the ΔΔCt method. Three independent biological replicates were used, and three technical replicates were performed per sample. The QPCR primers used in this study include:

Zmiz1 primer: Fwd: GTCAGCAACCATGTGTTCCACC, Rev: GCCAGTTGGTGTTCATCTGCCG
Tnc primer: Fwd: TTTGCCCTCACTCCCGAAG, Rev: AGGGTCATGTTTAGCCCACTC
Cplx3 primer: Fwd: AAGGGGGACGGAGACAAGT, Rev: CTGTGCATCTCGCTCCATCTT
Gria2 primer: Fwd: TTCTCCTGTTTTATGGGGACTGA, Rev: CTACCCGAAATGCACTGTATTCT

### Cortical morphology analysis

Cortical thickness was determined by measuring the shortest distance from pia to the border of the cortical plate and white matter in DAPI stained sections.

For layer thickness, sections were immunolabeled for either layer 6 marker Tbr1 (Abcam ab31940, rabbit anti-Tbr1, 1:500) or layer 5 marker Ctip2 (Abcam ab18465, rat anti-Ctip2). Layer 6 thickness measurements were taken tracing a straight line from the bottom border to the top of the Tbr1+ labeled layer, in parallel with the radial orientation of the cortex. Layer 5 thickness was similarly measured in the Ctip2+ layer. Upper layer thickness was measured from the top border of the Ctip2+ layer to the pia. All distance measurements were performed using Neurolucida software “Quick Measure Line” tool (Neurolucida, MBF Bioscience).

Three measurements were taken per section. Three sections per brain were analyzed. All measurements correspond to the motor cortex.

### Behavioral analysis

Marble Burying test was performed following standard protocol, as described in (38). Briefly, each mouse was removed from their home cage and placed in a clean cage with extra bedding (5cm depth) and 20 marbles arranged on top of the bedding in a 5 x 4 grid. The mice were placed in the corner of the cage on the right and closest to the experimenter. A lid was placed on the cage, and the mouse was left for 30 minutes. This experiment took place in a quiet, dark room designated for behavioral tests. After 30 minutes, each mouse was removed from the experimental cage and returned to their home cage. The unburied marbles were counted by two experimenters. Marbles were considered to be buried if more than 2/3 of the marble was beneath bedding. A total of 61 adult mice were used, 29 Zmiz1-KO and 52 control mice.

### Statistical analysis

Z-scores and fold changes were calculated following standard methods.
Z-scores (Z) = (x - µ)/σ
where, x = read count of a gene
µ = mean of read count for a gene across all sample
σ = standard deviation of read count for a gene across all sample

Fold changes were calculated as per DESeq2 standard:

We used RNA-seq differential expression tool which utilizes DESeq2 algorithm to calculate the fold change and differential analysis of count data as upregulated or downregulated genes. Detailed workflow of DESeq2 and code can be found in (92) as used by Illumina basespace differential expression tool.

We used unpaired two-tailed Student’s t-tests or one-way ANOVA followed by Tukey *post hoc* test for statistical comparison for behavioral, qPCR, and cortical thickness analysis. The same sizes and specific test used for each analysis is indicated in the figure legends. Values are represented as means ± SEM. Statistical significance is noted by asterisks, (* p<0.05, ** p<0.01).

### Publicly available resources used in the paper

Molecular Logic of Cellular Diversification in the Mammalian Cerebral Cortex (13). (https://singlecell.broadinstitute.org/single_cell/study/SCP1290/molecular-logic-of-cellular-diversification-in-the-mammalian-cerebral-cortex).

Allen Brain Atlas (https://mouse.brain-map.org/).

PsychENCODE (14), Human brain development (http://development.psychencode.org/#).

Tabula Muris (15) (https://tabula-muris.ds.czbiohub.org/).

UniProt Variants Viewer (www.uniprot.org/uniprotkb/Q9ULJ6/variants-viewer).

MobiDB, a database of protein disorder and mobility annotations (20) (https://mobidb.org/).

SFARI gene-scoring (https://gene.sfari.org/).

STRING database (50) (https://string-db.org/).

BioGRID 4.4 (51) (https://thebiogrid.org/).

DeCoN: Genome-wide Analysis of *In Vivo* Transcriptional Dynamics during Pyramidal Neuron Fate Selection in Neocortex (18).

Atlas of mRNA translation in humans (52) (https://smorfs.ddnetbio.com).

The mRNA translation landscape in the synaptic neuropil (53). (https://public.brain.mpg.de/dashapps/localseq/).

Factorbook (27, 28), epigenetic profile (https://factorbook.org/).

Synaptic Gene Ontologies, SynGO (54) (https://syngoportal.org/#).

### Software/resources usage details

Molecular Logic of Cellular Diversification in the Mammalian Cerebral Cortex (13). (https://singlecell.broadinstitute.org/single_cell/study/SCP1290/molecular-logic-of-cellular-diversification-in-the-mammalian-cerebral-cortex).

Go to the link -> explore -> In search genes and find plots-> distribution -> Plot type to Box plot.

Allen Brain Atlas (https://mouse.brain-map.org/).

Go to the link -> Enter Gene Name -> select experiment 74988259 -> Zmiz1 expression Box plot.

PsychENCODE (14), Human brain development (http://development.psychencode.org/#) Go to the link -> Search Data -> mRNA-seq, all regions -> type Zmiz1 and search -> download ZMIZ1_ENSG00000108175.PDF.

Go to the link -> Search Data -> mRNA-seq, NCX regions -> type Zmiz1 and search -> download ZMIZ1_ENSG00000108175.PDF.

Tabula Muris (15) (https://tabula-muris.ds.czbiohub.org/).

Go to the link -> go to Visualization section -> select FACS method, Brain Non-myeloid Tissue, and Zmiz1 Gene -> scroll down and select Violin Plot to visualize Zmiz1 expression.

UniProt Variants Viewer (www.uniprot.org/uniprotkb/Q9ULJ6/variants-viewer).

Go to the link (take straight to Zmiz1, for other gene, search in Search tab) -> select Likely pathogenic or pathogenic and Uncertain significance in the Variant viewer.

MobiDB, a database of protein disorder and mobility annotations (20) (https://mobidb.org/).

Go to the link -> search Zmiz1 -> select Q9ULJ6 Accession.

SFARI gene-scoring (https://gene.sfari.org/).

Go to the link -> Search Zmiz1 in Search SFARI Gene tab -> Select ZMIZ1.

STRING database (50) (https://string-db.org/).

Go to the link -> Go to SEARCH -> Search Zmiz1/Homo Sapiens -> select confidence in settings.

BioGRID 4.4 (51) (https://thebiogrid.org/).

Go to the link -> Search Zmiz1, Homo sapiens -> Go to network to visualize the Zmiz1 network.

DeCoN: Genome-wide Analysis of *In Vivo* Transcriptional Dynamics during Pyramidal Neuron Fate Selection in Neocortex (18).

DeCoN dataset classified significant gene expression profiles into 20 distinct patterns of gene expression. The clusters were then divided into 4 distinct groups: CPN cluster (5 clusters), ScPN cluster (5 clusters), CThPN cluster (5 clusters), and cell type independent cluster (5 clusters). To access the CPN, SCPN, and CThPN clusters with high specificity and stringency (DeCoN **Figure 3B**), we extracted gene lists from Clusters 5, 0, and 10 for CPN neuron subtype, Cluster 16, 18, and 11 for SCPN neuron subtype, and Cluster 6,9, and 2 for CThPN neuron subtype. These genes were then correlated with the P7 RNAseq DEGs list.

Atlas of mRNA translation in humans (52) (https://smorfs.ddnetbio.com).

Go to the link -> go to the Expression -> search for Zmzi1 ensembl gene ID (ENSG00000108175).

The mRNA translation landscape in the synaptic neuropil (53). (https://public.brain.mpg.de/dashapps/localseq/).

Go to the link -> go to Explore -> go to Translatome -> enter Zmiz1 in gene names tab and search -> export the MA plot.

Go to the link -> go to Explore -> go to Transcriptome -> enter Zmiz1 in gene names tab and search -> export the MA plot.

Factorbook (27, 28), epigenetic profile (https://factorbook.org/).

Go the link -> search Zmiz1 in Search Human TFs tab -> go to epigenetic profile.

Synaptic Gene Ontologies, SynGO (54) (https://syngoportal.org/#).

Go the link -> go the “Your gene list” -> input your gene list, select brain expressed, add a name in description -> start analysis -> export data for the SynGO annotated genes from your list.

### Figures tool

Figures and graphics were made using GraphPad Prism version 9.5.1 for Windows (http://www.graphpad.com) or BioRender (www.biorender.com). Venn diagrams were made using InteractiVenn (94).

## Data availability statement

The datasets presented in this study can be found in online repositories. The names of the repository/repositories and accession number(s) can be found in the article/supplementary material.

## Ethics statement

Ethical approval was not required for the studies involving humans because is not required for use of publicly available cell line datasets. The studies were conducted in accordance with the local legislation and institutional requirements. The human samples used in this study were acquired from Publicly available datasets from ENDODE, Allen Brain Institute, and SFARI databases. Written informed consent to participate in this study was not required from the participants or the participants’ legal guardians/next of kin in accordance with the national legislation and the institutional requirements. The animal study was approved by Tulane University Institutional Animal Care and Use Committee (IACUC). The study was conducted in accordance with the local legislation and institutional requirements.

## Author contributions

RK: Conceptualization, Data curation, Formal analysis, Investigation, Methodology, Visualization, Writing – original draft, Writing – review & editing. AT: Writing – review & editing, Data curation, Formal analysis, Investigation, Methodology. AT: Data curation, Writing – review & editing, Formal analysis, Investigation, Methodology. SMM: Funding acquisition, Project administration, Resources, Supervision, Writing – review & editing. MJG: Conceptualization, Data curation, Funding acquisition, Project administration, Resources, Supervision, Visualization, Writing – original draft, Writing – review & editing.
